# Study of the corrosion behavior of N80 and TP125V steels in aerobic and anoxic shale gas field produced water at high temperature

**DOI:** 10.1186/s13065-024-01225-z

**Published:** 2024-06-26

**Authors:** Lincai Peng, Shaomu Wen, Hongfa Huang, Xi Yuan, Jiahe Huang, Yu He, Wen Chen

**Affiliations:** 1 Research Institute of Natural Gas Technology, PetroChina Southwest Oil and Gasfield Company, Chengdu, Sichuan 610213 China; 2 National Energy R&D Center of High Sulfur Gas Exploitation, Chengdu, Sichuan 610213 China; 3 High Sulfur Gas Exploitation Pilot Test Center, China National Petroleum Corporation, Chengdu, Sichuan 610213 China; 4 PetroChina Southwest Oil and Gasfield Company, Chengdu, Sichuan 610051 China; 5 School of Chemical Engineering and Technology, Sun Yat-sen University, Zhuhai, Guanggong 519082 China; 6 Shunan Gas Mine, PetroChina Southwest Oil and Gasfield Company, Luzhou, Sichuan 646001 China

**Keywords:** Localized corrosion, Shale gas, N80 and TP125V steels, High temperature

## Abstract

In this study, the corrosion behavior of N80 and TP125V steels was delved firstly into produced water from shale gas fields containing CO_2_-O_2_. Moreover, the localized corrosion of these steels was investigated to elucidate the effects of aerobic and anoxic on steel corrosion. The results indicated that the corrosion rates of N80 and TP125V steels under aerobic conditions were lower compared to those in the presence of CO_2_-O_2_. Specifically, at temperature of 100 °C and with dissolved oxygen (DO) concentration of 4 mg/L in the CO_2_-O_2_ environment, the N80 and TP125V steels exhibited the highest corrosion rate, with values of 0.13 mm/y and 0.16 mm/y, respectively, as determined by specific weight loss measurements. Conversely, these rates decreased to 0.022 mm/y and 0.049 mm/y under aerobic conditions. Furthermore, severe localized corrosion of N80 and TP125V steels with a DO concentration of 4 mg/L was also observed in the CO_2_-O_2_ environment. Finally, it was evident that pitting corrosion is the predominant type of corrosion affecting N80 and TP125V steels in the produced water from shale gas fields.

**Supplementary Information:**

The online version contains supplementary material available at 10.1186/s13065-024-01225-z.

## Introduction

Shale gas, as a cleaner and more efficient form of natural energy, is at the forefront of oil and gas exploration and has been a hot topic of research in recent years [1, 2]. The estimated amount of recoverable shale gas is more than 30 × 10^12^ m^3^ in China, of which the Sichuan Basin has the highest reserves [3]. As the demand for shale gas increases, the current challenge is how to improve shale gas recovery efficiency. The safety running of pipeline steel is the primary factor in guaranteeing shale gas exploitation. Nevertheless, wellbores and gathering pipelines often suffer from severe corrosion due to the harsh environment in shale gas wells [4, 5]. Especially once the wellbore fails due to corrosion, it leads to the shutdown of gas wells for repairs, greatly restricting the high-efficiency exploitation of shale gas resources [6]. The corrosion of steel especially pitting corrosion is the principal reason influencing the mechanical properties of pipelines and their service life [7]. However, the corrosion mechanism of steel is also indefinable but depends on the environmental conditions.

Corrosion is closely related to material types and environmental conditions [8]. Carbon steel has been commonly applied in oil and gas fields because of its favorable mechanical properties as well as low costs [9]. The production tubing utilized in shale gas wells is commonly fabricated from materials such as N80 and TP125V steel grades [10]. However, carbon steel has a low corrosion resistance, and localized corrosion is commonly found [11]. Many factors have a deep influence on steel corrosion, such as temperature, pressure, dissolved oxygen, quality of produced water, and microorganisms [12–16]. Jiang et al. [17] discovered severe corrosion of mild steel in a shale gas gathering condition, and microbiological corrosion connected with under-deposit corrosion (UDC) made a big contribution to steel corrosion. The environments downhole in shale gas wells are complex, following the high pressure, high temperature, a high salt solution, and the maximum temperature can exceed 100 ℃ [18, 19]. Temperature as one of the important parameters that can directly influence the corrosion rates of steel, and there is an extreme value for steel corrosion rates as the increase of the temperature in a sealed environment [20].

Furthermore, hydraulic fracturing in shale gas wells can introduce dissolved oxygen (DO), which is also a significant factor in the corrosion of steel materials [21, 22]. DO interacts with iron in an aqueous medium to produce Fe_2_O_3_ [23]. Compared with the condition containing only CO_2_, the cathodic reaction rate could be accelerated, and reduce the formation probability of FeCO_3_ due to the conversion of FeCO_3_ into Fe_2_O_3_ in the presence of O_2_ [24]. Previous studies mainly focus on the influence of CO_2_, microorganisms, temperature, and Cl^−^ on steel corrosion [25, 26]. However, the impact of DO on the corrosion behavior of steel in a shale gas environment remains uncertain. The presence of oxygen in a CO_2_-saturated test solution has been shown to alter corrosion behavior and mechanisms [27]. Tang et al. observed that the introduction of DO in the supercritical CO_2_ phase can expedite the corrosion of steel, resulting in the formation of dual-layer corrosion products due to the presence of a small amount of DO [28]. In a similar vein, Li et al. [29]reported that DO could reduce the corrosion rate of N80 steel by promoting the formation of a protective FeCO_3_ layer under supercritical CO_2_ conditions.

In our previous studies, we found that the corrosion of TP125V and N80 steel was most extensive under conditions of 100 °C and saturated CO_2_ [30]. Consequently, this work focuses on the corrosion behavior of these two steels in a shale gas field produced water as the change of DO was deeply studied in this work, using weight loss, surface analysis, and electrochemical measurements, which provides insight into the possible influence of aerobic and anoxic shale gas environments on steel corrosion.

## Experimental

### Steel specimens

N80 and TP125V steels, commonly applied as the material for downhole tubing in the shale gas field, were used to explore the corrosion behavior, and their chemical compositions are shown in Table S1. The C content was determined using a Carbon-Sulfur Analyzer instrument (CS-3000, NCS Testing Technology Co., China), while the other elements were measured by Atomic Absorption Spectroscopy (iCE 300, Thermo Fisher Scientific, USA). Steel specimens were applied in the experiment of weight loss and surface morphologies with the size of 10 mm × 30 mm × 3 mm. Electrochemical experiments, including open-circuit potential (OCP), electrochemical impedance spectroscopy (EIS), and potentiodynamic polarization studies, were conducted using working electrodes with an area of 1 cm^2^ under stagnant. The samples were consistently polished with 400#, 600#, and 1200# silicon carbide abrasive papers, rinsed with ultrapure water, acetone, and anhydrous ethanol in series, following sterilization of more than 30 min by ultraviolet (UV) light.

### Test solution

In this work, the test solution was prepared based on the chemical components of the shale gas field produced water from Sichuan Changning Natural Gas Development Co., Ltd., and the chemical components of the artificial shale gas field produced water were as shown in Table S2. To simulate the natural shale gas produced water, the field used drainage aid of 0.1 wt%, drag reducer of 0.1 wt%, and biocide of 0.05 wt% which were added to the test solution. The drainage aid, drag reducer and biocide, obtained from Chengdu Nengte Technology Development Co., Ltd., with respective product numbers CT5-12 A, CT1-20D, and CT10-4B, serve as the principal materials for hydraulic fracturing gas extraction in shale gas wells in the Sichuan-Chongqing region. The experiments were divided into two parts. For the first part, the concentration of dissolved oxygen was 2, 4, and 6 mg/L, respectively, adjusted by sparging CO_2_ gas to investigate the influence of DO on steel corrosion. For the second part, the corrosion study of steel was conducted in an aerobic condition at 60℃ and 100 ℃ to instigate the influence of temperature on steel corrosion.

### Weight loss

After a specific time of testing, all specimens applied were taken out and were slightly washed with a pickling solution containing corrosion inhibitors. Then the washed specimens were further rinsed with distilled water, acetone, and absolute ethyl alcohol, respectively. Subsequently, all specimens were dried using N_2_. The weight loss was calculated based on the change in the specific mass of specimens. The following equation was used to calculate the corrosion rates of different steel specimens.

1 $$CR=\frac{8.76\times 1{0}^{4}\times ({M}_{1}-{M}_{2})}{At\rho }$$

Where *CR, A, t* and *ρ* are corrosion rate (mm/y), the working area of steel (cm^2^), testing time (h) and the density of steel (kg/m^3^), respectively. *M*_*1*_ and *M*_*2*_ are the specific mass of the specimens initially and after corrosion (g).

### Characterizations of surface films

The surface films of two steel specimens caused by corrosion under different test conditions were conducted by scanning electron microscopy (SEM, JSM-IT200, JEOL, Japan), and their chemical compositions were measured by X-ray diffractometer (XRD) (Ultima IV, Rigaku, Japan) and energy disperse spectroscopy (EDS) (JSM-IT200, JEOL, Japan). Before SEM observation, a thin gold film coated on the specimen aims to increase the quality of the images. The corrosion morphologies of bare specimens were observed by a three-dimensional microscope (Leica DVM6, Germany) to characterize the localized corrosion.

### Electrochemical measurements

Electrochemical tests in this work were conducted based on a three-electrode system via a CS350 electrochemical workstation, while steel specimens were the working electrode, Ag/AgCl electrode, and Pt plate corresponding to the reference and counter electrodes, respectively. OCP was scanned at a scan rate of 0.5 mV/s to achieve a stable state, and EIS was performed subsequently by applying sinusoidal voltage signal of 10 mV corresponding to the frequency range of 10^5^ ~ 10^− 2^ Hz. Potentiodynamic polarization curves with a potential range of -250 mV to + 350 mV vs. corrosion potential at a potential sweep rate of 0.5 mV/s. Zview2 and Cview2 software (Scribner, Inc.) were used to analyze the measured impedance and polarization data, respectively. All of the tests were repeated at least three times to ensure the reproducibility of experimental data.

## Results and discussion

### Effect of DO concentration on the corrosion of N80 and TP125V steels at 100℃

#### Corrosion rates based on weight loss

**Fig. 1:**
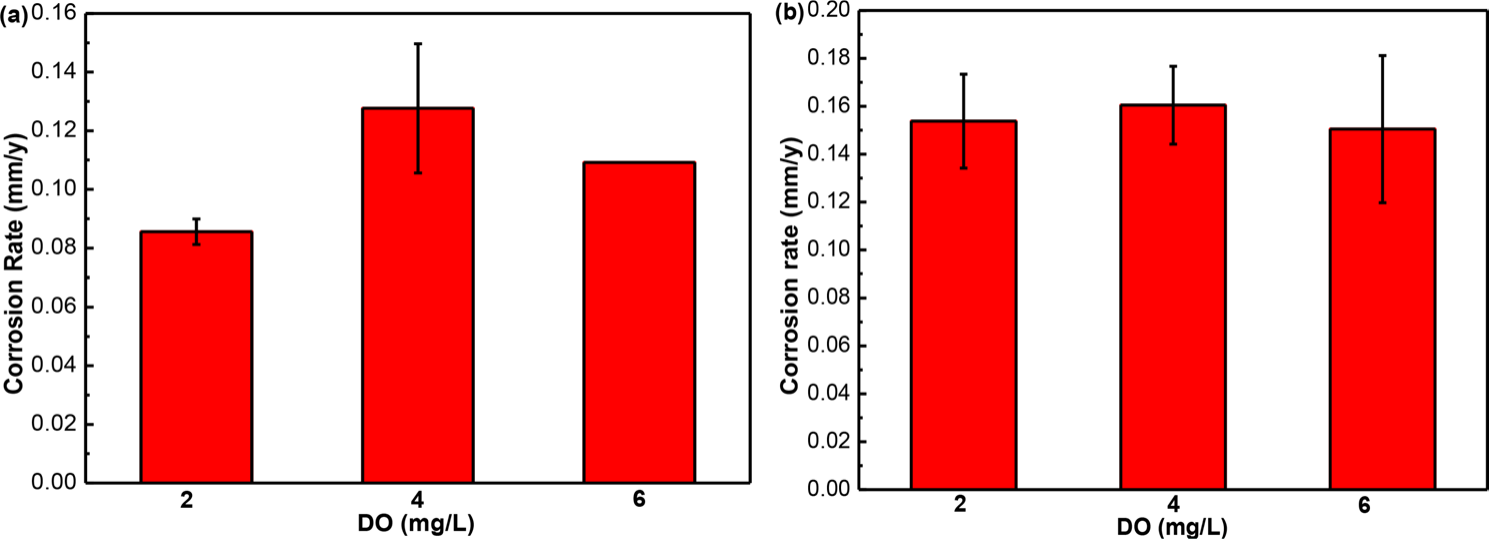
Corrosion rates of the specimens calculated from weight loss in artificial shale gas filed produced water containing the DO of 2, 4, and 6 mg/L at 100 ℃ after 3 days of testing: **(a)** N80 steel; **(b)** TP125V steel

The corrosion rates of N80 and TP125V steels calculated from the specific weight loss are depicted in Fig. 1 at 100 ℃ with different concentrations of DO and a test time of 3 d. As can be seen from Fig. 1, N80 and TP125V steels both have the biggest corrosion rates at the DO of 4 mg/L corresponding to the values of 0.13 and 0.16 mm/y respectively. Therefore, the changes in DO have an influence on steel corrosion in shale gas field conditions. The existence of O_2_ can change the components and structure of corrosion products thus then causing the change in steel corrosion behavior [31]. At this intermediate concentration, there is enough dissolved oxygen to promote the formation of corrosion products, such as oxides, which can enhance the rate of metal dissolution. However, at higher concentrations of DO, the formation of a more protective oxide layer can inhibit further corrosion, leading to a lower corrosion rate. Furthermore, it is also found that the corrosion rates of TP125V steel at 100 ℃ are slightly higher than those of N80 steel, which may be due to the composition of the material and its unique morphological characteristics [30].

#### Corrosion product morphology analysis

**Fig. 2:**
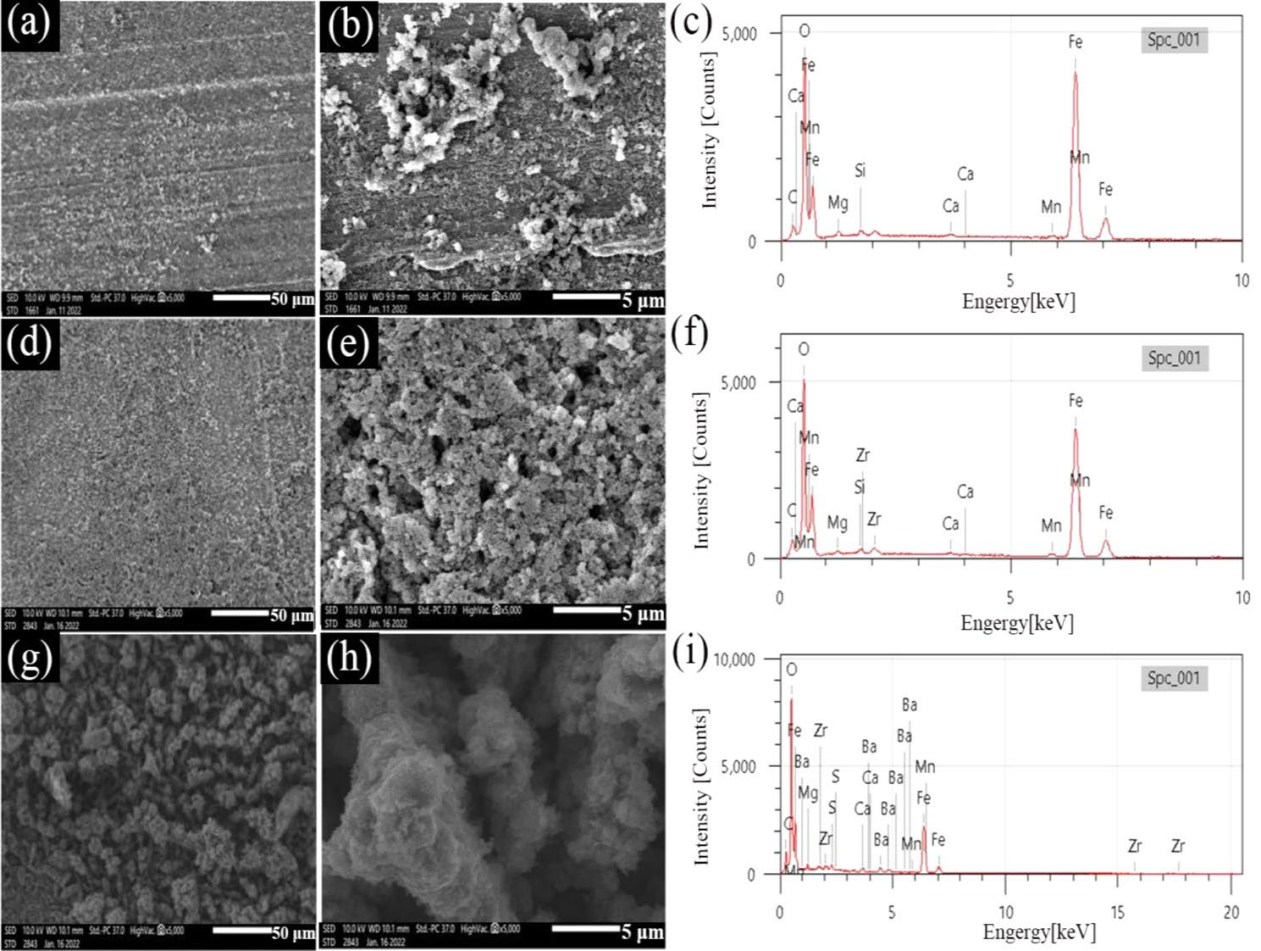
SEM images of surface films on N80 steel and the corresponding EDS after 3 days of testing at 100 ℃ in shale gas field produced water with different concentrations of DO: (**a**, **b** and **c**) 2 mg/L; (**d**, **e**, and **f**) 4 mg/L; (**g**, **h** and **i**) 6 mg/L

SEM images of the surface morphologies of N80 steel and the corresponding EDS after 3 d of testing at 100 ℃ in artificial shale gas field produced water with different concentrations of DO are presented in Fig. 2. There is a thin corrosion product film for the specimen with DO of 2 mg/L (Fig. 2a), and the polish lines and some particles of corrosion products are also seen (Fig. 2a and b). The appearance of polish lines suggests slight corrosion. When the DO increases to 4 mg/L and 6 mg/L, corrosion products on the surface of the specimen have noticeably increased and there are also some larger particles covering the surface of the specimen, which further indicates that the corrosion should be severe. A loose and porous corrosion product film appears (Fig. 2d and e) with DO of 4 mg/L which is conducive to the acceleration of steel corrosion due to the easy diffusion and transfer of corrosive ions, such as Cl^−^ [32]. As the DO further increases to 6 mg/L, a similar porous film structure is also found but the size of corrosion product particles is small (Fig. 2g and h). The smaller corrosion product particles and the dense structure can inhibit the erosion of corrosive ions, leading to a reduced corrosion rate. From the EDS analysis results, the dominating elements in corrosion products consist of C, O, Ca, Mg, Fe, and Cr. (Fig. 2c, f and i, and Table S3, and the contents of these elements under different test conditions are similar. Therefore, the primary corrosion products are FeCO_3_ and iron oxides on account of the coexistence of O_2_ and CO_2_ [33, 34].

**Fig. 3:**
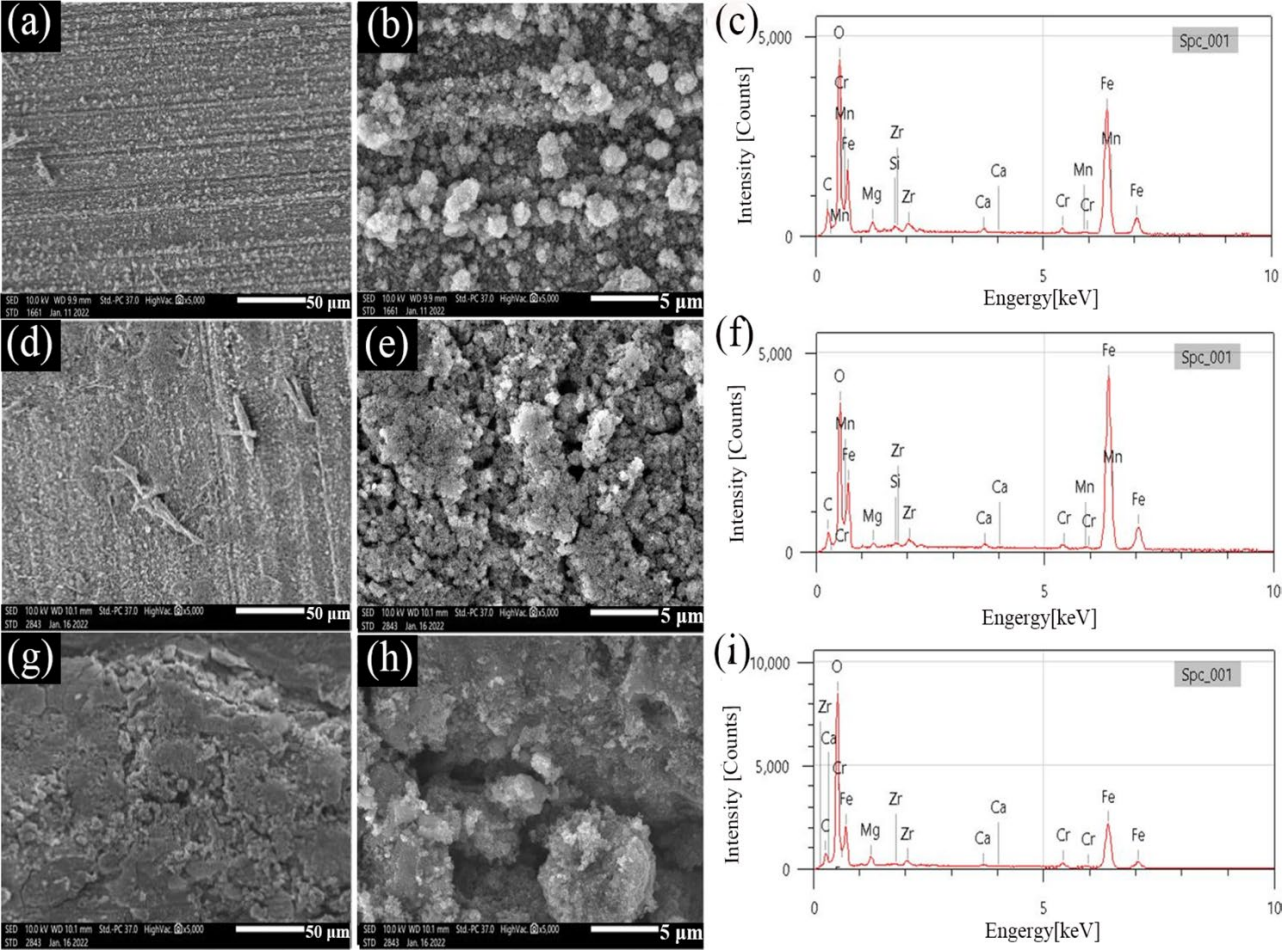
SEM images of surface films on TP125V steel and the corresponding EDS after 3 days of testing at 100℃ in artificial shale gas field produced water with different concentrations of DO: (**a**, **b** and **c**) 2 mg/L; (**d**, **e**, and **f**) 4 mg/L; (g, h and i) 6 mg/L

Figure 3 presents the SEM images of the surface morphologies of TP125V steel and the corresponding EDS after 3 d of testing at 100 ℃ in shale gas field produced water with different concentrations of DO. For TP125V steel, the surface morphologies are similar to N80 steel when the DO are 2 and 4 mg/L (Figs. 2 and 3), and the polish lines as well as the porous corrosion product films both can be observed. This also further explains the reason for the higher corrosion rate with a DO of 4 mg/L. Referring to Fig. 2b, the greater amount of corrosion products (Fig. 3b) also indicates that the corrosion of TP125 is more severe than that of N80 when the DO is 2 mg/L. When the DO is 6 mg/L, the denser corrosion product film can be recognized (Fig. 3g and h) and can inhibit the erosion of corrosive ions. This is also the reason for the decrease in the corrosion rate. The EDS analysis results also demonstrate that the elements in corrosion products include C, O, Fe, Ca, and Mg (Fig. 3c, f, i, and Table S4, also corresponding to the corrosion products of FeCO_3_ and iron oxides.

#### Analysis of the bare corrosion morphology of steels

**Fig. 4:**
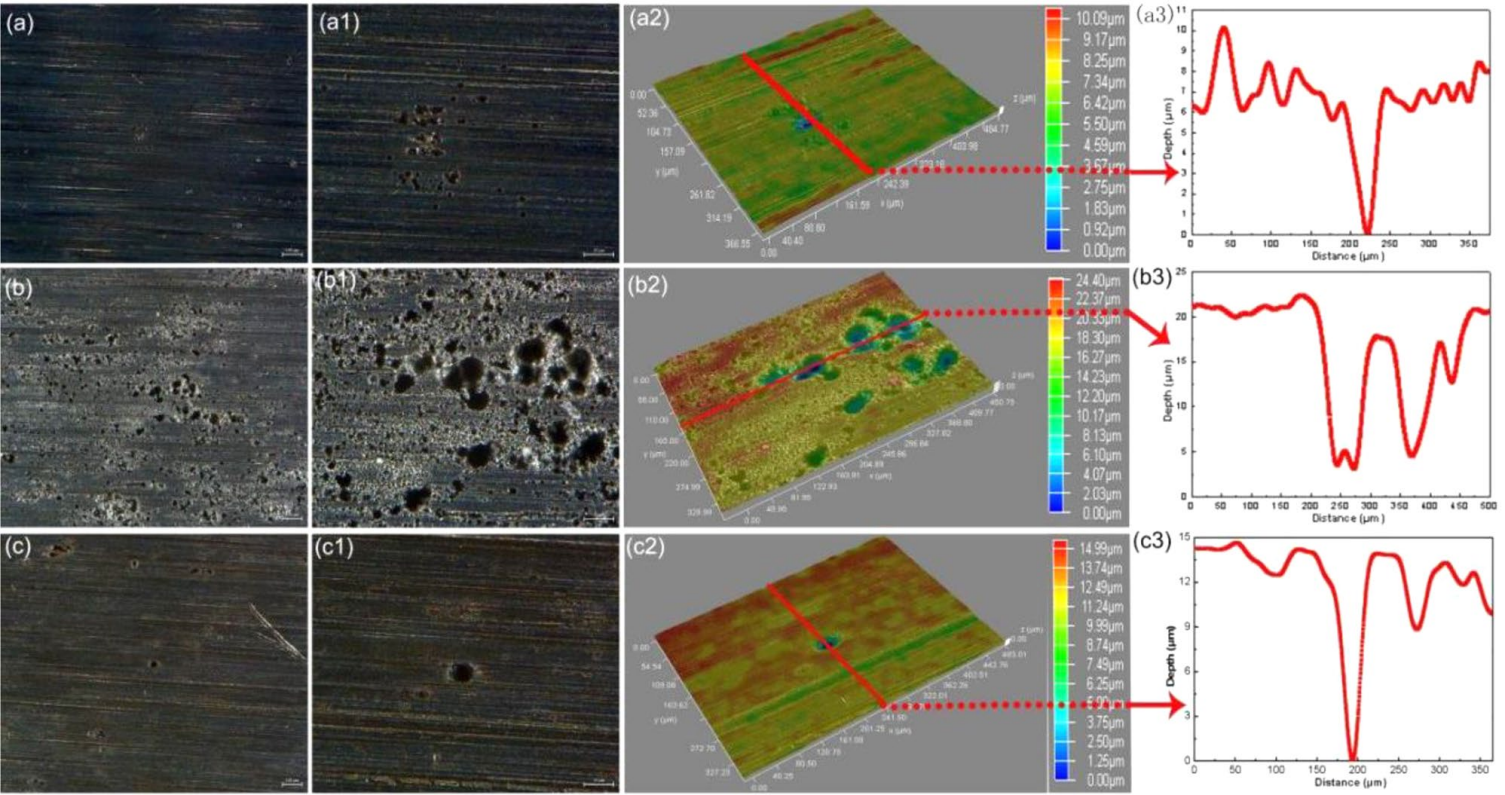
The bare corrosion morphologies of N80 steel without corrosion products after 3 days of testing at 100℃ in shale gas field produced water with different concentrations of DO: (a, a1 and a2) 2 mg/L; (b, b1, and b2) 4 mg/L; (c, c1, and c2) 6 mg/L

The bare corrosion morphologies of N80 steel after removing the surface corrosion products with a test time of 3 d at 100 ℃ are depicted in Fig. 4. From the overall point of view, the specimens suffer from different degrees of localized corrosion under different test conditions. For the specimen with DO of 2 mg/L, the polish scratches on the sample surface are still clearly visible, and some small and shallow corrosion pits can be observed with a maximum depth of about 10.09 μm (Fig. 4a-a2). When the DO increases to 4 mg/L, serious pitting corrosion on steel surface having a maximum depth of about 24.40 μm is observed (Fig. 4b-b2). However, both the density and depth of corrosion pits decrease for the specimen with DO of 6 mg/L, with the maximum depth of the corrosion pits reaching approximately 14.99 μm (Fig. 4c-c2). The test results of bare surface morphologies are in accordance with the corrosion rates (Fig. 1a), the corrosion of N80 steel is significantly enhanced when DO is 4 mg/L.

The bare corrosion morphologies of TP125V steel without corrosion products after 3 d are presented in Figure S1. From Figure S1 a-a2, the corrosion is slight with the DO of 2 mg/L, but some small areas corresponding to the black color can be attacked by corrosion. However, severe pitting corrosion is observed on steel specimens with the DO of 4 mg/L, and the maximum depth of corrosion pits is about 45.42 μm (**Figure **S15b-b2). When the DO furtherly increases to 6 mg/L, the corrosion of steel has an apparent mitigation and pitting corrosion has a maximum depth of about 26.93 μm (**Figure **S1 5c-c2). Similarly, these results are consistent with the corrosion rate findings presented in Fig. 1b. It can be concluded that the appearance of DO in the test solution has a deep influence on the corrosion process, and the uniform and pitting corrosion is more serious when the DO is 4 mg/L. Furthermore, the corrosion rate of TP125V steel has exceeded N80 steel in artificial shale gas field produced water. The generation of pitting corrosion can derive from the addition of field-used drainage aid, drag reducer, and biocide which have a corrosion inhibition effect on steel corrosion [12]. During the exploitation of oil and gas fields, large amounts of corrosion inhibitors are widely applied but pitting corrosion dominates one of the main corrosion types [35–37].

### Corrosion behavior of N80 and TP125V steels under aerobic and anoxic conditions

#### Corrosion rates derived from weight loss

**Fig. 5:**
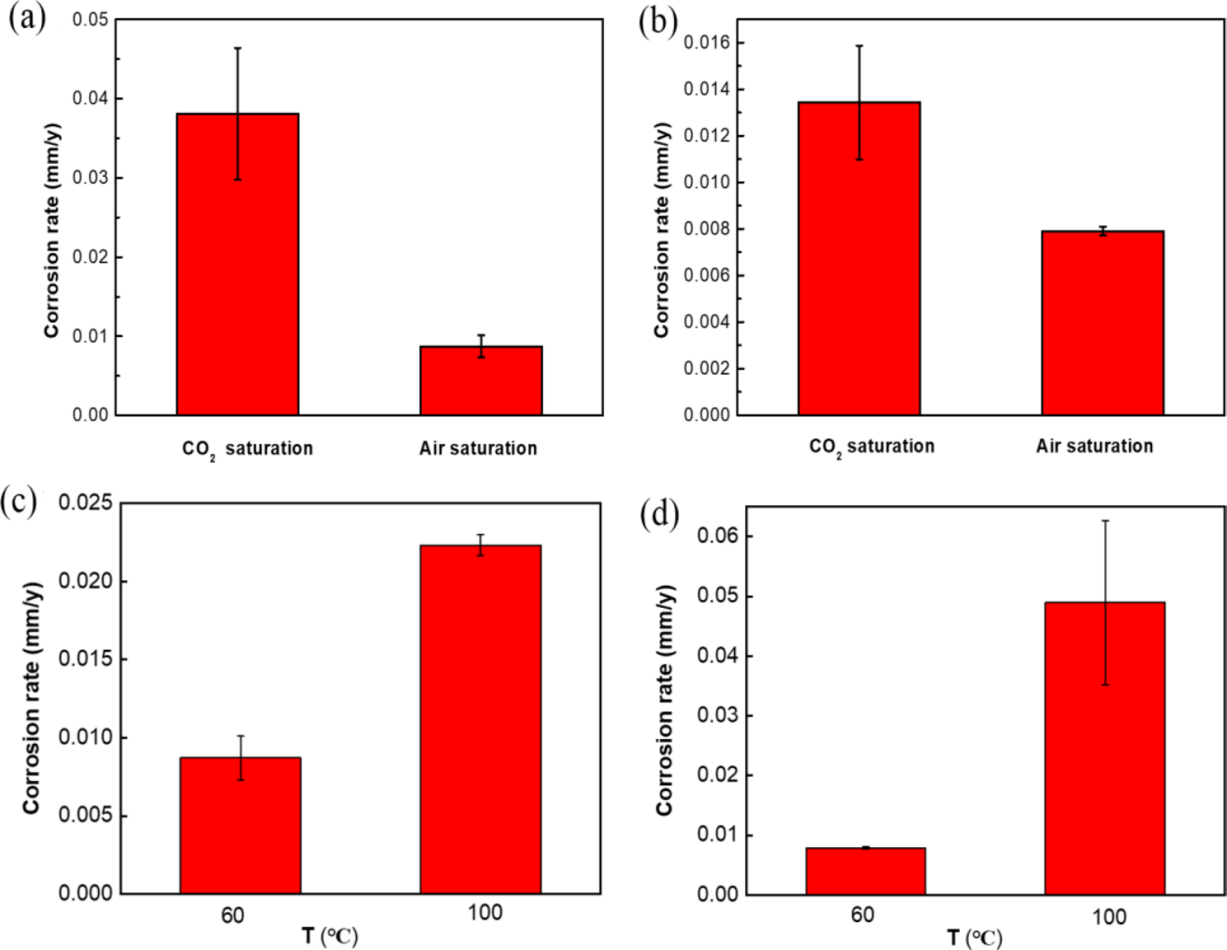
Corrosion rates of N80 steel **(a)** and TP125V steel **(b)** calculated from weight loss after 14 days of testing in CO_2_ saturation and air saturation in air-saturated artificial shale gas field produced water at 60℃; corrosion rates of N80 steel **(c)** and TP125V steel **(d)** calculated from weight loss after 14 days of testing in air-saturated artificial shale gas field produced water at 60 and 100 ℃

Figure 5 depicts the uniform corrosion rates of N80 and TP125V steels calculated from weight loss after 14 d of testing in CO_2_ saturated and in air-saturated artificial shale gas field produced water at 60℃ and in air-saturated artificial shale gas field produced water at 100 ℃. In the CO_2_-saturated environment, the corrosion rate of N80 steel reaches 0.038 mm/y, whereas in the air-saturated environment, the corrosion rate is only 0.009 mm/y, significantly lower than in the CO_2_-saturated environment (Fig. 5a). A similar result is observed for TP125V. The corrosion rate of TP125V steel is 0.013 mm/y in the CO_2_-saturated environment, while the corrosion rate is merely 0.008 mm/y in the air-saturated environment (Fig. 5b). This substantial reduction in the corrosion rate in the air-saturated environment compared to the CO_2_-saturated environment further indicates that CO_2_ has a greater promoting effect on metal corrosion than O_2_. Thus, it is evident that the corrosion rates of both steels in the anoxic condition are significantly higher than those in the air-saturated environment. This finding suggests that in shale gas environments, CO_2_ corrosion is more critical to consider than oxygen corrosion. The increase in temperature can accelerate the corrosion rates of steel belongs to the kinetics of electrochemical corrosion. Therefore, temperature is one of the considerable factors influencing steel corrosion in shale gas conditions. There is a low corrosion rate for N80 steel at 60 ℃ with a value of 0.009 mm/y, but the value increases to 0.022 mm/y at 100 ℃ (Fig. 5c). For TP125V steel, the corrosion rates of the specimen reach 0.008 and 0.049 mm/y at 60 and 100 ℃, respectively (Fig. 5d). Therefore, it can be concluded that the corrosion of N80 and TP125V steels has a little difference at a low temperature, but the corrosion of TP125V steel is more serious compared to N80 steel at a high temperature, i.e., TP125V steel has a lower corrosion resistance under high-temperature conditions. Higher temperature can increase the migration of corrosive ions and accelerate both the anodic and cathodic reactions. Thus then, the corrosion rate on both steel increased as the temperature increased. Furthermore, it is also discovered that steel corrosion in the air-saturated test solution has a decrease compared to that in the CO_2_-O_2_ solution. Therefore, the individual oxygen corrosion of pipeline steel in shale gas produced water does not need to be paid more attention.

#### Corrosion product morphology analysis

**Fig. 6:**
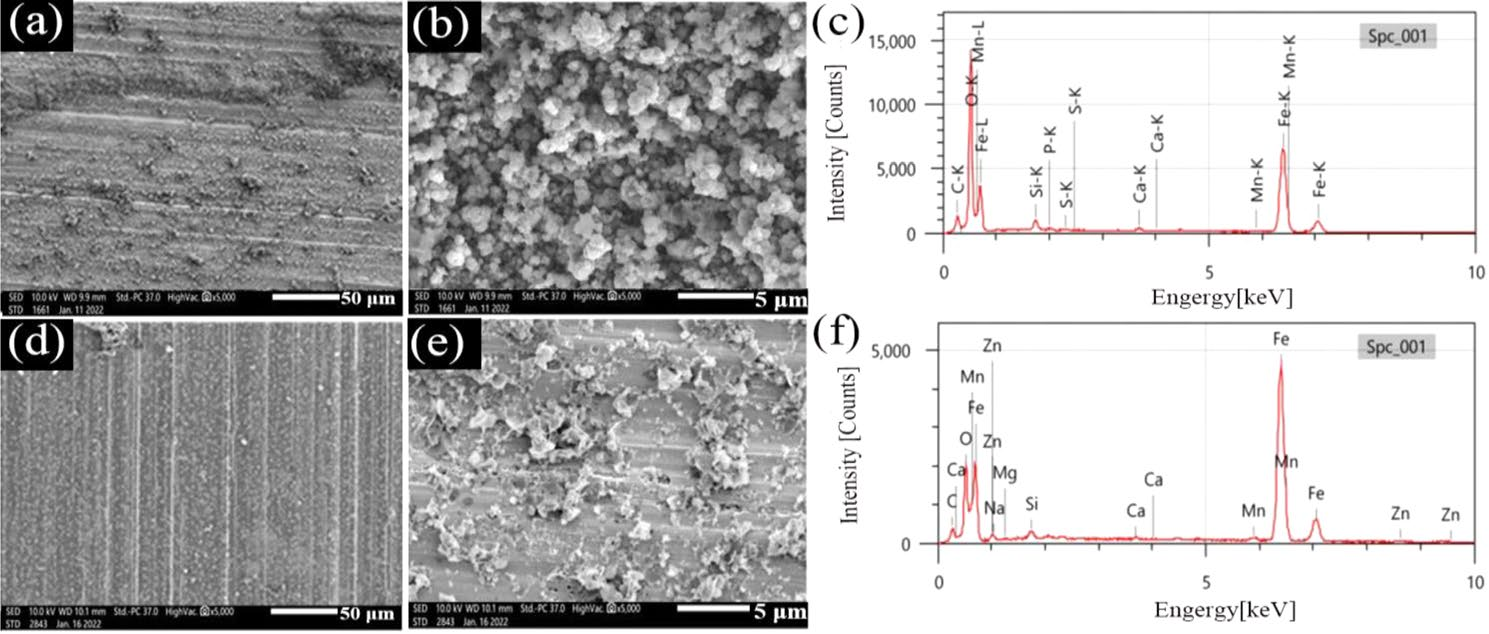
SEM images of surface films on N80 steel and the corresponding EDS after 14 days of testing at 60 ℃ and 100 ℃ in aerobic shale gas field produced water: (**a**, **b**, and **c**) 60 ℃; (**d**, **e**, and **f**) 100 ℃

**Fig. 7:**
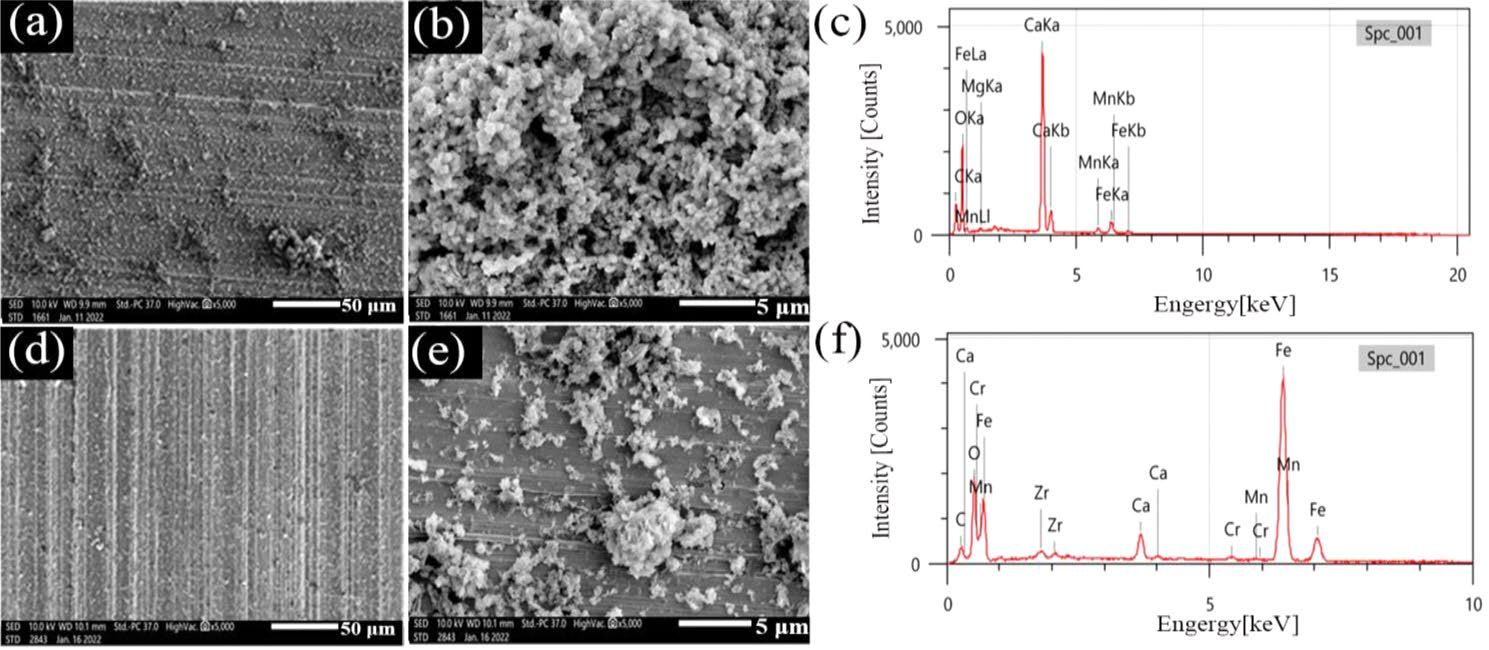
SEM images of surface films on TP125V steel and the corresponding EDS after 14 days of testing at 60 ℃ and 100 ℃ in aerobic shale gas field produced water: (**a**, **b**, and **c**) 60 ℃; (**d**, **e**, and **f**) 100 ℃

SEM images of surface films on N80 and TP125V steels and the corresponding EDS after 14 d of testing at 60 and 100 ℃ in aerobic shale gas field produced water are displayed in Figs. 6 and 7. For N80 steel, there is a thin film caused by corrosion on steel at 60 ℃ (Fig. 6a), and some agglomerate corrosion product particles cover on steel surface (Fig. 6a and b). When the temperature increases to 100 ℃, only little corrosion products are found (Fig. 6c and d), and some corrosion pits are also observed (Fig. 6d), suggesting a weak uniform corrosion. When the steel changes to TP125V steel, the morphologies of the surface film have a little change compared to N80 steel. Some loose corrosion products can also be found at 60 ℃, and the surface corrosion products have decreased when the temperature is 60 ℃ (Fig. 7d and e). Furthermore, EDS analysis results demonstrate that the main corrosion products focus on iron oxides (Tables S5 and S6), the typical products of oxygen corrosion.

SEM images in Figs. 6 and 7 show that the surface scratch lines of N80 and TP125V steels are easily recognized no matter whether the temperature is 60 or 100 ℃ which demonstrates that the uniform corrosion is not severe for steel specimens corresponding to the weight loss (Fig. 5). The key factor in this work causing a low corrosion rate is the adding of some chemicals with corrosion inhibition effect as stated above. Furthermore, steel corrosion rates in the aerobic condition are smaller than that in the CO_2_-O_2_ environment (Figs. 1 and 5). Therefore, the presence of CO_2_-O_2_ in the test solution can considerably promote steel corrosion but the corrosion will alleviate when only in the O_2_ environment.

#### XRD analysis results

**Fig. 8:**
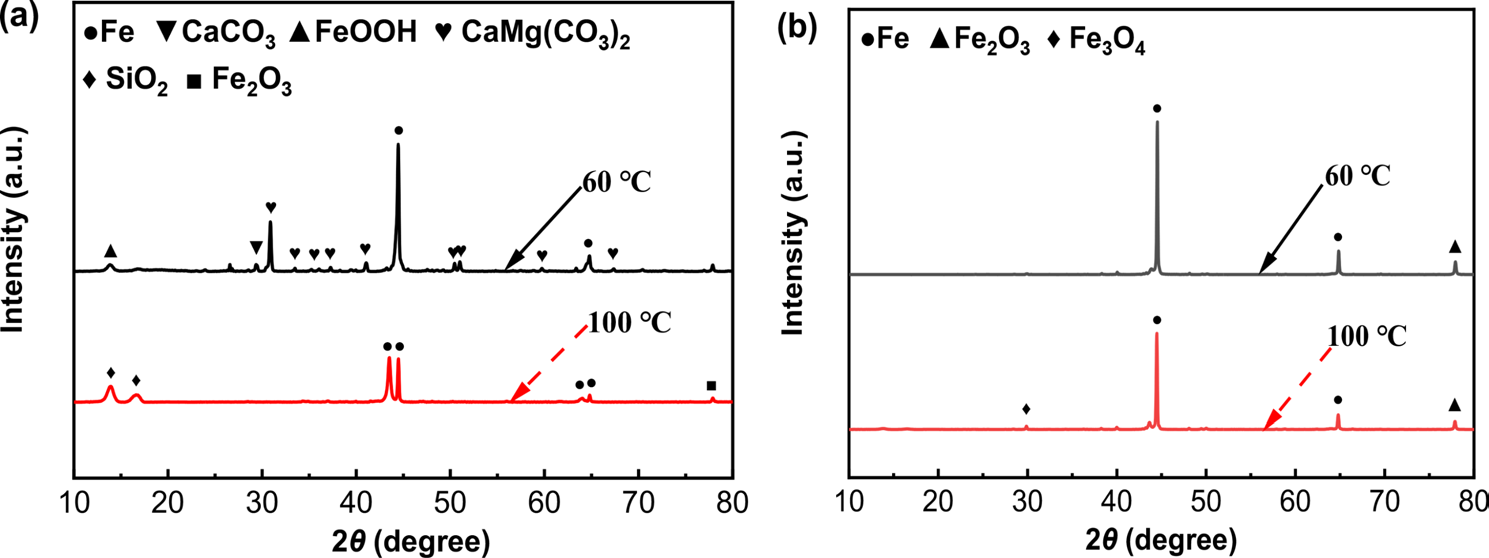
XRD analysis results of the corrosion products on N80 **(a)** and TP125V **(b)** steels after 14 days of testing at 60 and 100 ℃ in aerobic shale gas field produced water

Figure 8 shows the specific components of the corrosion products formed on N80 and TP125V steels after 14 d of testing at 60 and 100 ℃ in aerobic shale gas field produced water. As exhibited in Fig. 8a, the typical corrosion products of N80 steel at 60 ℃ are mainly composed of CaCO_3_, FeOOH, and CaMg(CO_3_)_2_, while Fe_2_O_3_ is dominated at 100℃. For TP125V steel, Fe_2_O_3_ and Fe_3_O_4_ dominate the primary corrosion products (Fig. 8b). The peak intensity of the corrosion products is low and Fe has a high peak in XRD spectra, which demonstrates that the contents of surface corrosion products are low corresponding to SEM images of surface films (Figs. 6 and 7).

#### Analysis of the bare corrosion morphology

**Fig. 9:**
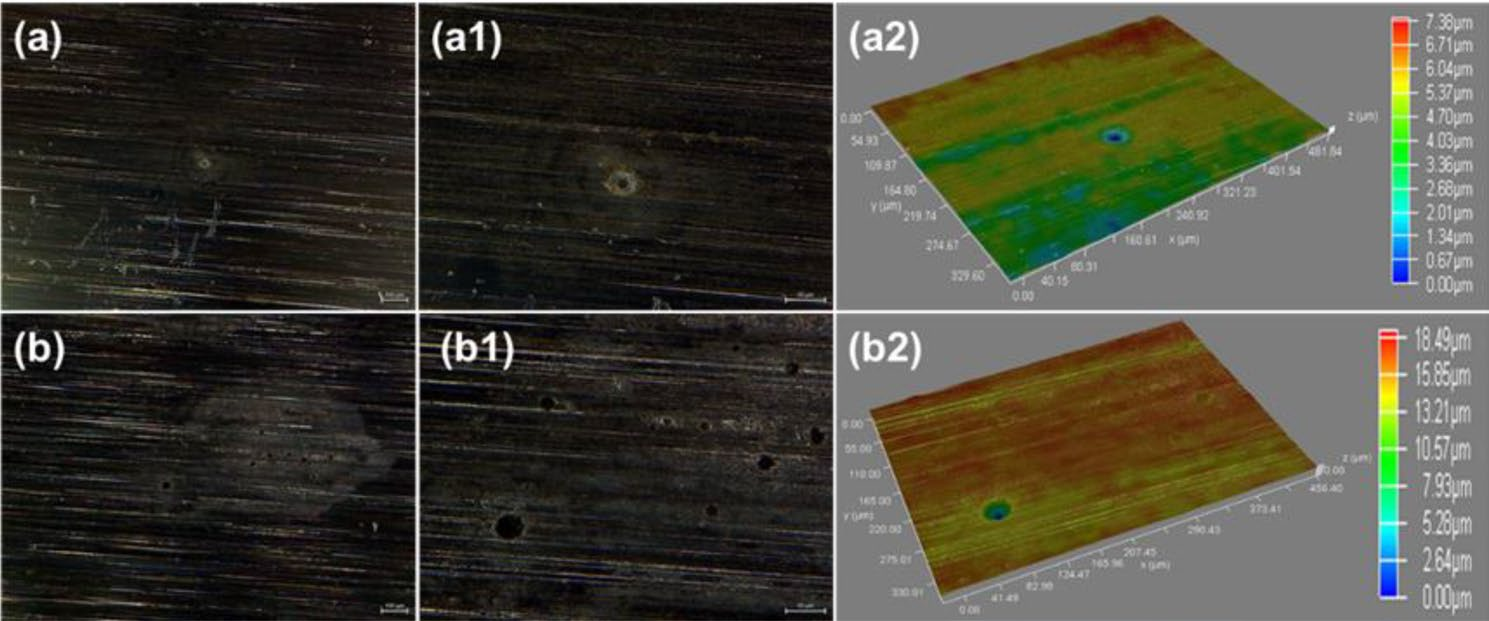
The bare surface corrosion morphologies of N80 steel after 14 days of testing at 60 and 100 ℃ in aerobic shale gas field produced water: (a-a2) 60 ℃, (b-b2) 100 ℃

**Fig. 10:**
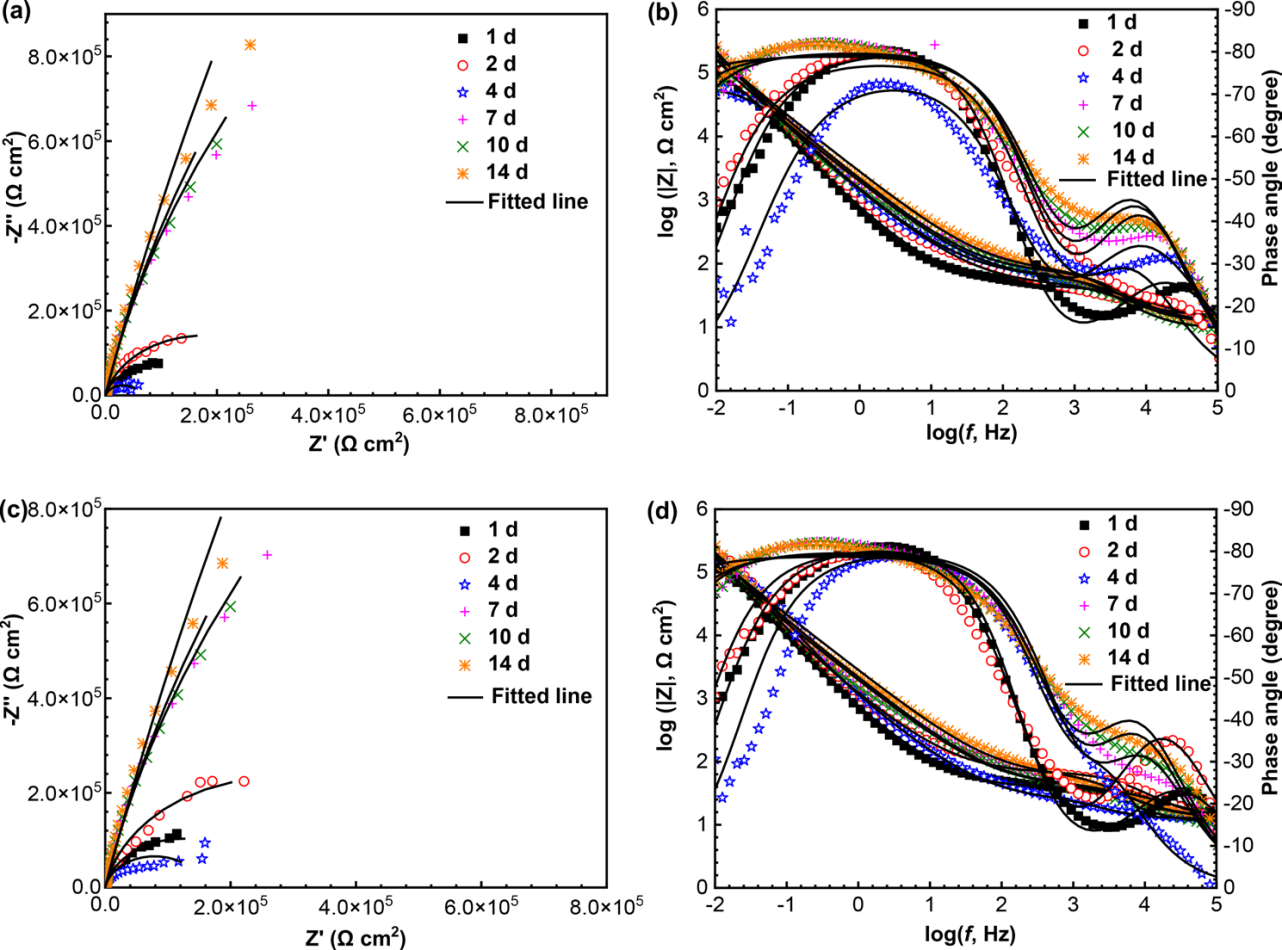
Nyquist and Bode diagrams of N80 (**a** and **b**) and TP125V (**c** and **d**) steels after 14 days of testing at 60 ℃ in aerobic shale gas field produced water

The bare surface corrosion morphologies of N80 and TP125V steels after 14 d of testing at 60 and 100 ℃ in aerobic shale gas field produced water are exhibited in Figs. 9 and 10. At 60 ℃, both the corrosion of N80 and TP125V steels is slight especially localized corrosion, and only some minute-sized corrosion pits are found (Fig. 9a-a2 and 10a-a2). When the temperature increases to 100 ℃, the density and depth of corrosion pits have a considerable increase compared with those at 60 ℃. For N80 steel, the maximum depth of pitting corrosion is about 18.49 μm at 100 ℃ (Fig. 9b2). However, the size of the corrosion pits of TP125V steel (Figure S2) is higher than those for N80 steel, and its maximum depth is about 20.69 μm (Figure S2 b2). Therefore, the localized corrosion of TP125V steel in aerobic shale gas field produced water at a high temperature is more serious compared with N80 steel, which is also in agreement with weight loss (Fig. 5). The formation of pitting corrosion can derive from the formation of some defects in corrosion inhibitor film as well as the erosion of Cl^−^ [38].

#### EIS analysis

The Nyquist and Bode diagrams of N80 and TP125V steels with a test time of 14 d at 60 ℃ in aerobic shale gas field produced water are shown in Fig. 10. To do the electrochemical analysis, 60 ℃ is chosen as a typical temperature. For N80 steel, the diameters of Nyquist plots have a gradual increase as time overall, but the impedance values change little after 7th d (Fig. 10a). Two time-constants are easily found in Bode plots, demonstrating the formation of a protective film limited the diffusion process of corrosive ions [39]. The two time-constants are formed initially on the first day. Therefore, the formed surface film cannot be the corrosion product film, but an adsorption film of corrosion inhibitor coming from the field used drag reducer, drainage aid and biocide. This agrees with the SEM observation in Fig. 9a and b, i.e., no apparent corrosion product film formation. A similar situation is also found in the Bode plots of TP125V steel (Fig. 10d). Furthermore, the impedance values of TP125V steel also have a similar tendency to N80 steel.

**Fig. 11:**
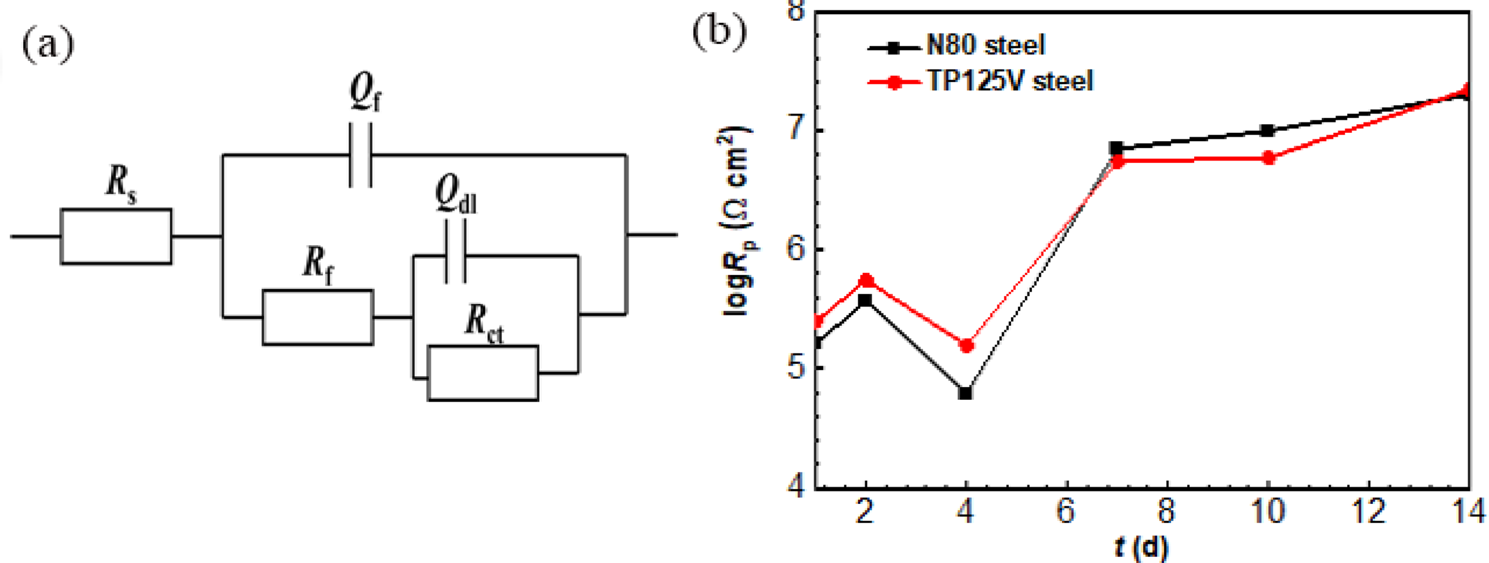
Equivalent circuit **(a)** used to fit EIS data and the corresponding fitted results of TP125V steel **(b)**, i.e., *R*_p_, the sum of *R*_f_ and *R*_ct_

EIS diagrams are fitted well based on the equivalent circuit presented in Fig. 11a, and the corresponding *R*_p_ values, the sum of *R*_f_ and *R*_ct_, i.e., the fitted results of EIS data are exhibited in Fig. 11b. *R*_p_ values are inversely proportional to steel corrosion rates. In the equivalent circuit, *R*_s_, *R*_f,_ and *R*_ct_ are assigned to the resistances of solution, surface film as well as charge transfer, while *Q*_f_ and *Q*_dl_ correspond to surface film and double-layer capacitance. From Fig. 11b, the changes of *R*_p_ values of N80 and TP125V steels with time have a similar law, but the *R*_p_ values of TP125V steel are higher than N80 steel before 7 d. There is a small difference in the *R*_p_ values of N80 and TP125V steels after 7 d of testing but the *R*_p_ values of TP125V steels are smaller overall. These demonstrate that the corrosion behavior of N80 and TP125V steels at 60 ℃ has a little difference and their corrosion difference is related to the test time.

#### Polarization curves

**Fig. 12:**
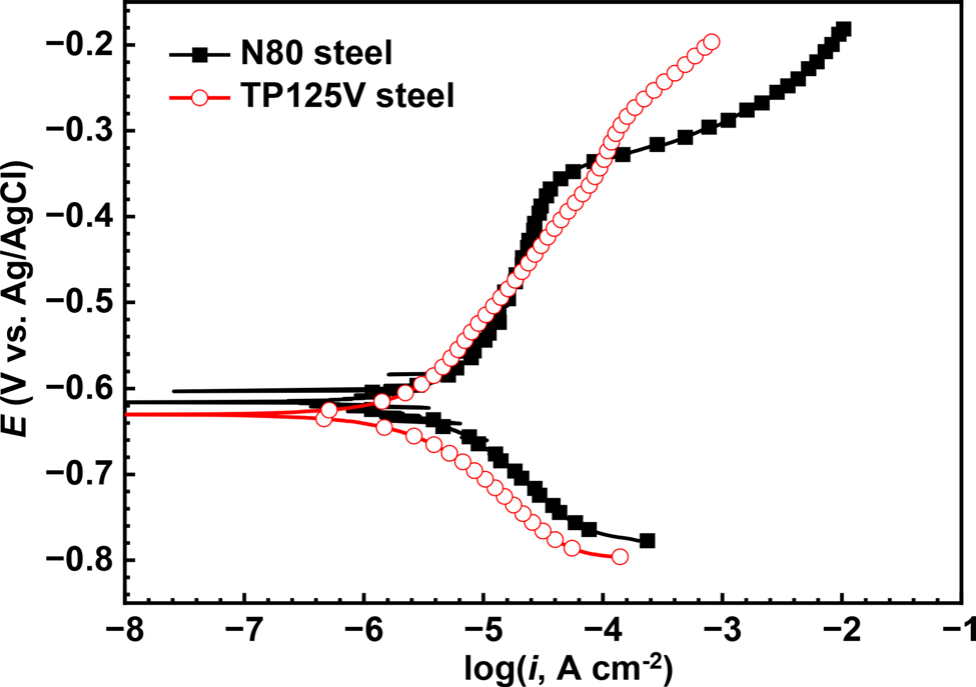
Polarization curves of N80 **(a)** and TP125V **(b)** steels after 14 days of testing at 60 ℃ in aerobic shale gas field produced water

Figure 12 presents the polarization curves of N80 and TP125V steels after 14 d of testing at 60 ℃ in aerobic shale gas field produced water, and the fitted electrochemical parameters including the anodic and cathodic Tatel slopes (*B*_a_ and *B*_c_), the corrosion potential (*E*_corr_), the corrosion current density (*I*_corr_) and the corrosion rate are exhibited in Table S7. The cathodic reaction of TP125V steel is inhibited while its anodic reaction is slightly enhanced compared with the N80 steel. The corrosion potential of TP125V steel has a little negative shift. However, the polarization curves of N80 and TP125V steels have a small difference. From the fitted electrochemical parameters in Table S7, the corrosion current densities of N80 and TP125V steels are close, and the values are 3.44 × 10^− 6^ and 2.40 × 10^− 6^ A/cm^2^, respectively. The values of corrosion current density show a positive correlation with steel corrosion rates. Therefore, it is also concluded that the corrosion behavior of N80 and TP125V steels at 60 ℃ are similar, and the corrosion rate of N80 steel is slightly higher than TP125V steel. Furthermore, a passivation phenomenon can be seen from the polarization curve of N80 steel, which is probably formed due to the corrosion product film.

## Conclusion

The introduction of DO in CO_2_-saturated artificial shale gas filed produced water has a deep influence on steel corrosion behavior, and N80 and TP125V steels both have the biggest corrosion rates with the DO of 4 mg/L at 100 ℃ corresponding to the values of 0.13 and 0.16 mm/y respectively. Serious localized corrosion is also found for N80 and TP125V steels at the DO of 4 mg/L, and the maximum depths of pitting corrosion are about 24.40 and 45.42 μm, respectively. When the DO is 2 and 6 mg/L, both the uniform and localized corrosion are slight. The increase in the content of DO leads to an intensification of corrosion to a certain extent, but further increases do not exacerbate corrosion, which can be relate to the characteristics of the corrosion products. Localized corrosion is the main corrosion type for N80 and TP125V steels deriving from the addition of some corrosion-inhibitive components.

N80 and TP125V steels have high corrosion rates in CO_2_-saturated test solution with values of 0.038 and 0.013 mm/y, and the values turn out to be 0.009 and 0.008 mm/y in air-saturated test solution at 60 ℃. It is evident that the corrosion rates of both steels in the anoxic condition are significantly higher than those in the air-saturated environment. Besides, the corrosion rates of N80 and TP125V steels with values of 0.022 and 0.049 mm/y at 100 ℃ are higher compared to their rates at 60 ℃, which indicates that temperature is also a key factor influencing the degree of oxygen corrosion. In addition, weight loss, the bare corrosion morphologies, and the analysis results of electrochemical measurements demonstrate that the corrosion difference between N80 and TP125V steels at 60 ℃ is small. Localized corrosion is also the typical corrosion type in aerobic conditions, and the main corrosion products are iron oxides, the typical products of oxygen corrosion. EIS data confirm the adsorption and formation of a corrosion inhibitor film on steel thus leading to slight uniform corrosion.

### Electronic supplementary material

Below is the link to the electronic supplementary material.

Supplementary Material 1

## Data Availability

All data generated or analyzed during this study are included in this published article.
