# Supplementary material for: Study of the corrosion behavior of N80 and TP125V steels in aerobic and anoxic shale gas field produced water at high temperature
Source: BMC Chem. 2024 Jun 26;18(1):117. doi: 10.1186/s13065-024-01225-z (PMC11210053; doi:10.1186/s13065-024-01225-z)
Supplement: Supplementary file 1 — Supplementary Material 1 [file 13065_2024_1225_MOESM1_ESM.docx]

Additional file

Study of the corrosion behavior of N80 and TP125V steels in aerobic and anoxic shale gas field produced water at high temperature

Lincai Peng^a,b,c^, Shaomu Wen^d^, Hongfa Huang^a,b,c^, Xi Yuan^a,b,c^, Jiahe Huang^e^, Yu He^f^, Wen Chen^a,b,c,*^

^a^Research Institute of Natural Gas Technology, PetroChina Southwest Oil and Gasfield Company, Chengdu, Sichuan, 610213, China

^b^National Energy R&D Center of High Sulfur Gas Exploitation, Chengdu, Sichuan, 610213, China

^c^High Sulfur Gas Exploitation Pilot Test Center, China National Petroleum Corporation, Chengdu, Sichuan, 610213, China

^d^PetroChina Southwest Oil and Gasfield Company, Chengdu, Sichuan, 610051, China

^e^School of Chemical Engineering and Technology, Sun Yat-sen University, Zhuhai, Guanggong, China

^f^Shunan Gas Mine, PetroChina Southwest Oil and Gasfield Company, Luzhou, Sichuan, 646001, China

*Correspondence: chen.wen@petrochina.com.cn


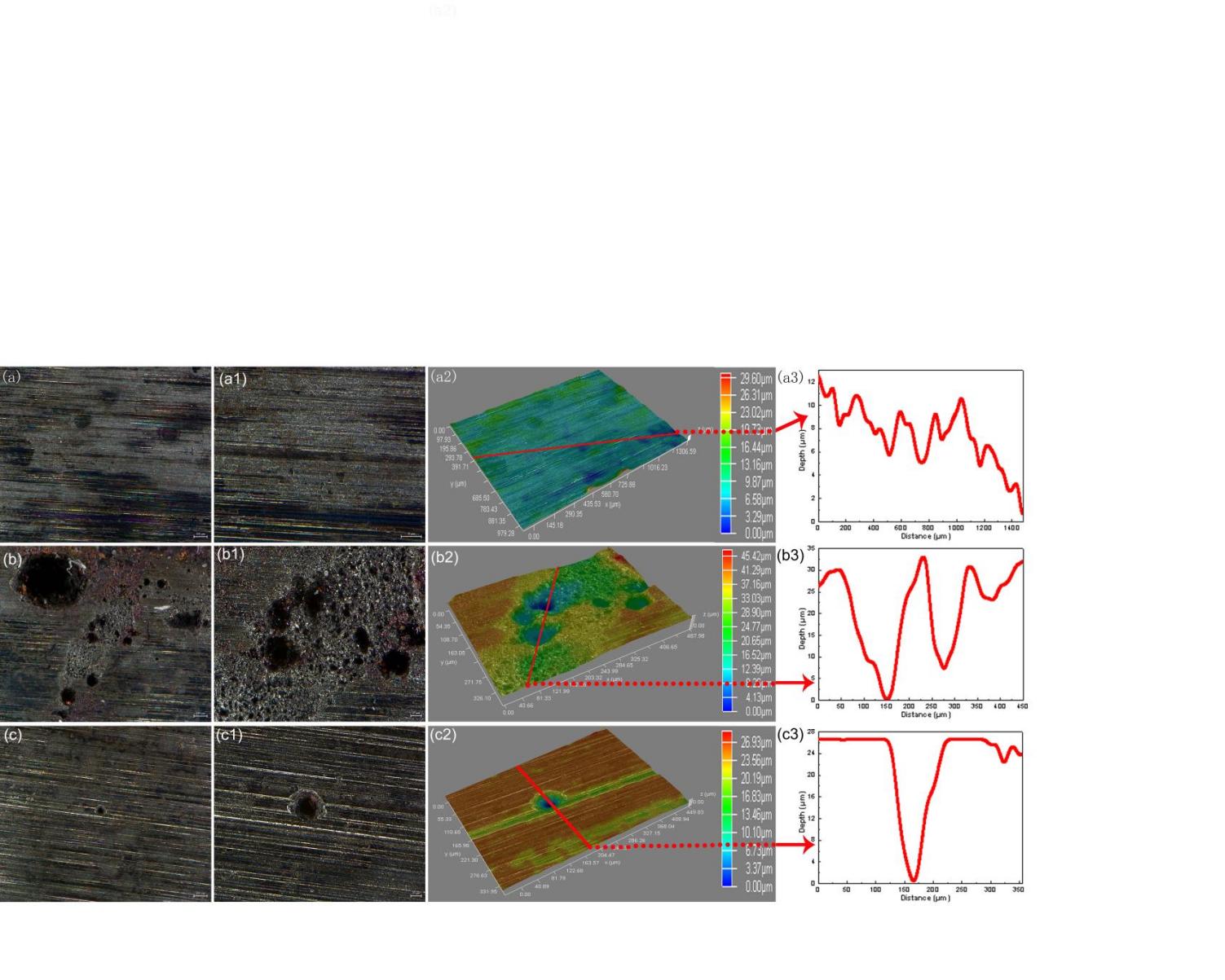


**Figure S1** The bare corrosion morphologies of TP125V steel without corrosion products after 3 days of testing at 100 ℃ in shale gas field produced water with different concentrations of DO: (a, a1 and a2) 2 mg/L; (b, b1, and b2) 4 mg/L; (c, c1, and c2) 6 mg/L.


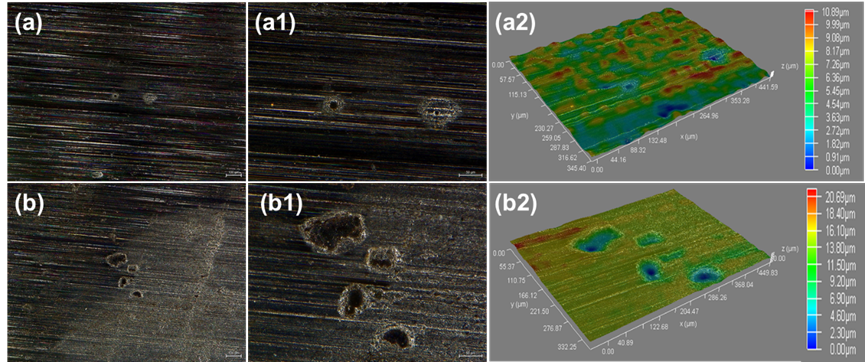


**Figure S2** The bare surface corrosion morphologies of TP125V steel after 14 days of testing at 60 and 100 ℃ in aerobic shale gas field produced water: (a-a2) 60 ℃, (b-b2) 100 ℃.

**Table S1** Chemical composition of N80 and TP125V steels (wt.%) used in this work.

|  | C | Si | Mn | P | S | Cr | Ni | Cu | V | Mo | Fe |
| --- | --- | --- | --- | --- | --- | --- | --- | --- | --- | --- | --- |
| N80 steel | 0.424 | 0.384 | 1.56 | 0.009 | 0.007 | 0.006 | <0.003 | 0.06 | 0.121 | <0.004 | Balance |
| TP125V steel | 0.288 | 0.314 | 0.606 | 0.009 | 0.004 | 0.872 | <0.003 | 0.093 | 0.099 | 0.639 | Balance |

**Table S2** Chemical composition of produced water from Changning shale gas production platform of China (mg/L).

|  | K^+^ | Na^+^ | Mg^2+^ | Ba^2+^ | Ca^2+^ | Sr^2+^ | Cl^-^ | SO_4_^2-^ | HCO_3_^-^ |
| --- | --- | --- | --- | --- | --- | --- | --- | --- | --- |
| Concentration of ions (mg/L) | 183.44 | 4961.67 | 30.45 | 34.82 | 63.12 | 33.29 | 8478.47 | 707.86 | 847.88 |

**Table S3** The analysis results of EDS spectra of N80 steel in Fig. 2 with different concentrations of DO.

| Elements (wt.%) | C | O | Ca | Fe | Si | Mn | Mg | Cr | S |
| --- | --- | --- | --- | --- | --- | --- | --- | --- | --- |
| 2 mg/L | 12.11 | 33.21 | 0.53 | 48.36 | 0.49 | 0.42 | 1.55 | 1.09 | - |
| 4 mg/L | 8.80 | 33.34 | 0.40 | 54.11 | 0.36 | 0.60 | 0.60 | - | - |
| 6 mg/L | 8.21 | 32.19 | 0.93 | 48.83 | - | 0.84 | 0.90 | 5.17 | 1.06 |

**Table S4** The analysis results of EDS spectra of TP125V steel in Fig. 2 with different concentrations of DO.

| Elements (wt.%) | C | O | Ca | Fe | Si | Mn | Mg | Cr |
| --- | --- | --- | --- | --- | --- | --- | --- | --- |
| 2 mg/L | 12.11 | 33.21 | 0.53 | 48.36 | 0.49 | 0.42 | 1.55 | 1.09 |
| 4 mg/L | 8.04 | 23.84 | 0.44 | 63.38 | 0.28 | 0.51 | 0.75 | 0.81 |
| 6 mg/L | 6.73 | 34.89 | 0.80 | 50.72 | - | - | 1.90 | 2.74 |

**Table S5** EDS analysis results of the corrosion products of N80 steel after 14 days of testing at 60 and 100 ℃.

| Elements (wt.%) | C | O | Ca | Fe | Si | Mn | Mg | Zn | Na |
| --- | --- | --- | --- | --- | --- | --- | --- | --- | --- |
| 60℃ | 8.21 | 13.64 | 0.30 | 74.08 | 0.91 | 1.15 | 0.29 | 0.78 | 0.65 |
| 100℃ | 6.82 | 5.92 | 0.34 | 85.47 | - | 1.45 | - | - | - |

**Table S6** EDS analysis results of the corrosion products of TP125V steel after 14 days of testing at 60℃ and 100℃.

| Elements (wt.%) | C | O | Ca | Fe | Si | Mn | Cr | Mg | Zr |
| --- | --- | --- | --- | --- | --- | --- | --- | --- | --- |
| 60 ℃ | 7.2 | 15.21 | 4.01 | 70.51 | - | 0.50 | 0.76 | - | 1.81 |
| 100 ℃ | 9.10 | 8.41 | - | 77.74 | 1.54 | 0.8 | 0.76 | 0.32 | - |

**Table S7** The fitted electrochemical parameters of the polarization curves of N80 steel and TP125V steels after 14 days of testing at 60 ℃ in aerobic shale gas field produced water.

|  | *B*_a_ (V dec^-1^) | *B*_c_ (V dec^-1^) | *E*_corr_ (V vs. Ag/AgCl) | *i*_corr_ (A/cm^2^) | corrosion rate (mm/y) |
| --- | --- | --- | --- | --- | --- |
| N80 steel | 0.176 | -0.077 | -0.619 | 3.44×10^-6^ | 0.040 |
| TP125V steel | 0.174 | -0.125 | -0.627 | 2.40×10^-6^ | 0.028 |
